# Mitochondrial Transfer in Cancer: A Comprehensive Review

**DOI:** 10.3390/ijms22063245

**Published:** 2021-03-23

**Authors:** Luca X. Zampieri, Catarina Silva-Almeida, Justin D. Rondeau, Pierre Sonveaux

**Affiliations:** Pole of Pharmacology, Institut de Recherche Experimentale et Clinique (IREC), Université Catholique de Louvain (UCLouvain), Avenue Hippocrate 57 Box B1.57.04, 1200 Brussels, Belgium; luca.zampieri@uclouvain.be (L.X.Z.); ana.dasilvaalmeida@studio.unibo.it (C.S.-A.); justin.rondeau@uclouvain.be (J.D.R.)

**Keywords:** cancer, cancer metabolism, mitochondria, mitochondrial transfer, tunneling nanotubes (TNT), oxidative phosphorylation (OXPHOS), tricarboxylic acid (TCA) cycle, reactive oxygen species (ROS), metastasis, chemoresistance

## Abstract

Depending on their tissue of origin, genetic and epigenetic marks and microenvironmental influences, cancer cells cover a broad range of metabolic activities that fluctuate over time and space. At the core of most metabolic pathways, mitochondria are essential organelles that participate in energy and biomass production, act as metabolic sensors, control cancer cell death, and initiate signaling pathways related to cancer cell migration, invasion, metastasis and resistance to treatments. While some mitochondrial modifications provide aggressive advantages to cancer cells, others are detrimental. This comprehensive review summarizes the current knowledge about mitochondrial transfers that can occur between cancer and nonmalignant cells. Among different mechanisms comprising gap junctions and cell-cell fusion, tunneling nanotubes are increasingly recognized as a main intercellular platform for unidirectional and bidirectional mitochondrial exchanges. Understanding their structure and functionality is an important task expected to generate new anticancer approaches aimed at interfering with gains of functions (e.g., cancer cell proliferation, migration, invasion, metastasis and chemoresistance) or damaged mitochondria elimination associated with mitochondrial transfer.

## 1. Introduction

Cancer is a metabolically heterogeneous disease. In particular, the balance between the rates of glycolysis (i.e., the conversion of glucose to lactate), the tricarboxylic (TCA) cycle and oxidative phosphorylation (OXPHOS) can be altered to match biomass production and energy needs. These metabolic changes are the result of multiple factors including genetic mutations, alterations of the tumor microenvironment (e.g., tumor oxygenation, extracellular pH, substrate availability) and interactions between adjacent cells/cell types.

Paradoxically, regardless if cells commit to OXPHOS or glycolysis as a primary mode of energy production, both pathways account for mitochondrial damage. Through the production of mitochondrial reactive oxygen species (mtROS) as a natural byproduct of OXPHOS, oxidative cancers can self-damage their mitochondrial DNA (mtDNA), mitochondrial membranes and component parts [1]. Conversely, even though glycolysis does not directly produce ROS, anaerobic glycolysis has also been shown to generate mtROS due to a partial oxidation of O_2_ to superoxide instead of water [2]. Hypoxia-inducible factor-1 (HIF-1), a key biomarker and regulator of glycolysis, is directly activated by ROS [3]. Moreover, anticancer therapies, such as alkylating chemotherapy and radiotherapy, also damage mtDNA through an oxidative/oxidative-like process [4,5]. In addition to superoxide, endogenously produced H_2_O_2_ can inflict further mitochondrial damage in its participation in the Fenton reaction, whereby cellular accumulation of Fe^2+^ catalyzes the production of the hydroxyl radical ^•^OH, a highly toxic ROS molecule with no natural scavenger [6]. Inside mitochondria, cytochrome c can potentiate this reaction by releasing free Fe^2+^ [7].

Since mtDNA encodes subunits of complexes of the electron transport chain (ETC), its integrity is correlated with OXPHOS efficiency. Cells can prevent mitochondrial oxidative damage through antioxidant enzymes belonging to the glutathione peroxidase (GPx), thioredoxin reductase, peroxiredoxin reductase, superoxide dismutase (SOD) and catalase (CAT) families [8,9]. Imbalances between oxidative stress and antioxidant defense can thus lead to increased mtDNA damage, creating single nucleotide polymorphisms (SNPs), insertions or deletions (indels). These mutations can be classified as homoplasmic when present in all mtDNA copies or heteroplasmic when present in only part of the copies [10].

mtDNA, already fragile in nature due to a lack of histones to protect from insults, can accumulate damage in a self-sustaining cycle: mtROS inflict mtDNA damage, which in turn produces less efficient mitochondria, which ultimately produce more mtROS. To protect cells against harm, mitochondrial integrity is stringently managed via mitophagy (the autophagic destruction of mitochondria) and mitochondrial biogenesis (the creation of new mitochondria) [9]. The onset of mitophagy is triggered by structural damages that disrupt mitochondrial function, while initiation of mitochondrial biogenesis is triggered by context-specific cellular demand. If mtDNA damage prevention and repair fail, cells can exchange copies of mtDNA using mitochondrial transfer, thus reintegrating functional mtDNA copies to restore mitochondrial duties. This is the topic of the present review.

## 2. Mechanisms of Mitochondrial Transfer

Mitochondrial transfer involves the incorporation of either mitochondrial genes or mitochondria themselves into a recipient cell. This phenomenon can lead to significant changes in the bioenergetic state of the host and/or to alterations related to cell differentiation, inflammatory processes, cell survival or even drug resistance. Mitochondrial transfer relies on the communication between a donor and a recipient cell and can be regulated by several structures, such as extracellular vesicles (EVs), tunneling nanotubes (TNTs) and gap junctions (GJ), among others [11]. Table 1 displays all studies involving mitochondrial transfer in cancer over the last 10 years. To note, mtDNA can persist, and the effects of transferred mitochondria can be active for at least 45 cell passages in vitro (135 days) [12]. Others demonstrated the maintenance of acquired phenotypes over at least 21 days [13].

### 2.1. Tunneling Nanotubes

The initial observations about the existence of structures termed “tunneling nanotubes” (TNTs) were made by Rustom et al. in 2004 [14]. TNTs were described as de novo formed structures between pairs of cells or complex cellular networks used as a way of transporting cellular components and signals. Before this discovery, similar structures with identical functional roles were already observed including cilia, filipodia and cytonemes [15,16,17,18,19].

#### 2.1.1. Formation of Tunneling Nanotubes

Since there are many different types of TNTs and TNT-like structures described in the literature (see references of Table 1) and due to the lack of specific TNT-markers, there is still some debate about the various types of TNTs [20].

**Figure 1:**
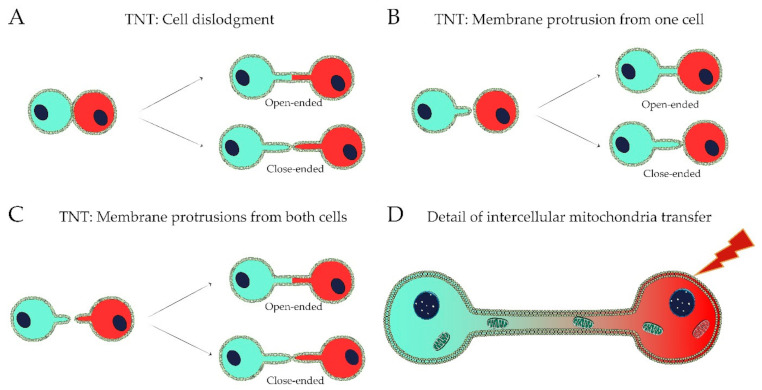
Mechanisms of formation of TNT and detail of intercellular mitochondria transfer. (**A**) TNTs can be formed by cell dislodgment, when cells are close together and move apart, creating TNTs that can be either open-ended or close-ended. (**B,C**) TNTs can occur by the extension of membrane protrusion(s) from one cell (**B**) or from both cells (**C**), and can be open-ended or close-ended. (**D**) Damage-inducing events can trigger the transfer of healthy mitochondria from healthy cells to damaged cells.

Two main mechanisms for the formation of TNTs have been discussed [20,24,41] (Figure 1A–D). The first mechanism is highly dependent upon cell mobility and occurs when cells are spatially proximal to each other and then diverge [42,43,44]. The movement of cells in opposite directions may lead to TNT rupture establishing close-ended or GJ connections. This process can also be temporally regulated, as a sustained intercellular contact for several minutes is needed for TNT formation [43]. The second mechanism occurs by the extension and fusion of membrane protrusions (MPs) containing actin filaments from the donor cell to the cell membrane of the target cell and does not rely on cell mobility or close contact [14,45]. The connection formed between cells can be open-ended through membrane fusion or GJ formation, or close-ended where the cargo needs to cross donor and recipient cell plasma membranes [24,33].

The mechanism of MP and TNT formation is largely associated with the interaction between a complex of proteins, including leukocyte specific transcript 1 (LST1), M-sec, Ras-related protein A (RalA) and the exocyst complex [45,46,47]. Schiller et al. [47] proposed a model for the formation of TNTs where LST1 recruits RalA to the plasma membrane and promotes its interaction with the exocyst complex, inducing actin polymerization and membrane complementation. In this model, M-sec acts as an inducer of TNT formation by interacting with LST1 and RalA.

Although molecular mechanisms driving TNT formation have been preliminarily elucidated using non-malignant models [48], more recent studies have also addressed this concept in cancer models. In a mesothelioma model, fascin, which is associated with initiation of cellular protrusions, distant metastasis and poor prognosis in advanced tumors [49], was observed over the length of TNTs. In addition, ezrin and zonula occludens-1 (ZO-1), which are involved in the organization of the actin cytoskeleton, were found at the site of TNT extrusion. Furthermore, E-cadherin, which is downregulated in invasive cancer cells and is an early independent marker of tumor progression, was also found to be minimally expressed during TNT formation [30]. Most recently, α-synuclein, a protein associated with Parkinson’s disease progression, was discovered to bind to migrating mitochondria in TNTs [37]. Aside from markers of cytoskeletal dynamics and rearrangement, the activation of the Akt-mTOR axis also triggers F-actin polymerization and TNT development [45]. In a highly malignant urothelial T24 model, an increase in the number of TNTs correlated with an upregulation of Akt and mTOR signaling compared to non-malignant urothelial RT4 cells, providing further evidence that this pathway is needed for TNT formation [33]. Finally, the expression of CD38, an immune cell marker regulating cell adhesion and signal transduction, was found to be correlated with TNT formation in a multiple myeloma model whereby increased CD38 expression facilitates mitochondrial transfer from bone marrow stromal cells (BMSCs) to primary human multiple myeloma cells [34]. CD38 expression blockade inhibited mitochondrial transfer, reduced tumor volume, and increased overall mouse survival.

Recent progress has made great strides surrounding the mechanisms underlying TNT propagation. Although the initial stimulus triggering TNT formation remains to be elucidated, cell stress, cell fate determination and changes in the microenvironment are all hypothesized to play central roles [20].

#### 2.1.2. Microtubules in Tunneling Nanotubes

In general, actin polymerization is paramount for TNT formation, as compounds disrupting actin functions damage TNT integrity [48,50,51]. However, some TNTs also contain microtubules and have been shown to be thicker in size [40,51]. The existence of TNTs with or without microtubules suggests that these structures have different biochemical roles. For example, co-culture of healthy and UV-damaged PC12 rat phaeochromocytoma cells showed that apoptotic signals were passed from damaged cells to heathy cells through thinner TNTs than TNTs used to carry mitochondria in the same cells [21]. It was also reported that thinner TNTs are responsible for the short-distance transport of mitochondria, while thicker microtubule-containing TNTs are necessary for longer-distance transport [21,52]. Although categorization of TNTs as “thinner” or “thicker” is suggested by some authors, it should also be noted that recent findings show that thicker TNTs can be composed by a group of several individual TNTs [53].

Studies in neuronal cells have identified a calcium-sensitive adaptor protein, mitochondrial Rho GTPAse 1 (Miro1), that, along with a group of accessory proteins Miro2, trafficking kinesin protein 1 (TRAK1), TRAK2 and myosin 19 (Myo19), facilitates mitochondrial movement through microtubules [13,54,55]. Furthermore, engineering MSCs to overexpress Miro1 increased their capacity as mitochondrial donors to epithelial cells, with enhanced therapeutic efficacy [47]. Conversely, in vitro models of leukemia, neuroblastoma and astrocytoma revealed that, although α-synuclein was necessary for the export of mitochondria through TNTs, Miro1 knockdown had no effect on either the density of mitochondria or the interaction between α-synuclein and mitochondria within TNTs [37]. The importance of microtubule mitochondrial transfer was also demonstrated in studies with acute lymphoblastic leukemia (ALL) cells, as agents that disrupted microtubule formation interrupted the process of cell death protection induced by mitochondria transfer [35].

#### 2.1.3. Tunneling Nanotube Trafficking

Although the process of mitochondrial transfer through TNTs is thought to be from donor to recipient cell, this is not always the case. Trafficking within TNTs can vary according to cell type, disease state and activation state (Table 1). For example, vesicles containing mitochondria are transported unidirectionally between neuronal cells [14] but bidirectionally between macrophages [51]. In some cases, mitochondrial transfer within TNTs can be a bidirectional process, but it is mainly unidirectional from healthy to damaged cells [21,50]. In studies performed with mesothelioma and benign mesothelial cell lines, there was an exchange of cytosolic components between mesothelioma cell lines or between benign mesothelial cell lines, but no evidence of exchange or TNT formation between malignant and benign cell populations [25]. In another study, physiological transfer of mitochondria occurred among MSCs and between MSCs and cancer cell lines, but not among cancer cells [26]. Furthermore, mitochondrial transfer occurred from bone marrow stromal cells (BMSCs) to acute myelogenous leukemia (AML) cells, but not to healthy hematopoietic cells [32]. Contrary to this, unidirectional transport was observed from malignant to non-malignant urothelial cell lines [33].

Interestingly, even when mitochondrial transport is bidirectional, the benefits that recipient cells receive may differ. For example, bidirectional mitochondrial transfer occurs between MSCs and vascular smooth muscle cells (VSMCs), but increased proliferation was only observed in MSCs [56]. Furthermore, both AML and ALL cells depend on mitochondrial transfer to reduce metabolic stress; while ALL cells export mitochondria to MSCs to reduce intracellular ROS, AML cells import mitochondria from MSCs to offset increased OXPHOS demand [36]. Together, these studies highlight the complex phenomena surrounding mitochondrial trafficking and directional transfer as a process that is cell- and context-specific.

### 2.2. Gap Junctions

Connexins are GJ proteins that facilitate the exchange of small molecules (less than 1 kD in size) between neighboring cells. As well as playing an important role in electrical and metabolic coupling between cells, many members of the connexin family have also been regarded as tumor suppressor genes [57]. In an in vivo model of LPS-treated lung disease, BMSCs were found to transfer mitochondria in microvesicles through GJ protein connexin 43 (Cx43) to nearby lung epithelium [58]. The authors noted that Cx43 was essential for mitochondrial transfer, as cells expressing dysfunctional Cx43 were not able to participate in this phenomenon.

### 2.3. Cell Fusion

Mitochondrial transfer can occur either by partial cell fusion via TNT formation or by complete cell fusion. For example, transfer of mitochondria was described upon complete fusion between MSCs and ischemic cardiomyoblasts [59]. However, mitochondria were also noted to be transferred via the formation of TNTs between these two cell types. Similarly, primary glioblastoma cells were found to take up mitochondria by engulfing tumor-activated stromal cells (TASCs), thereby sequestering TASC cellular components for themselves [38].

### 2.4. Artificial Mechanisms of Mitochondrial Transfer

Although most studies involving mitochondrial transfer are based on the physiological release and uptake of mitochondria, studies have also demonstrated that mitochondria can be transferred through artificial means. MitoCeption, a new tool designed to better understand artificially implanted mitochondrial dynamics, has been created to artificially transfer isolated mitochondria from MSCs to cancer cell lines [26]. This method can be a valuable tool to understand the effects of transferred mitochondria into recipient cells independently of other factors.

Another method of artificial isolated mitochondrial transfer employs a Pep-1-mediated delivery system [60,61]. Pep-1 is a member of the cell-penetrating peptide family that can deliver biologically active peptides and proteins into cells via interaction with cell membranes. The feasibility of mitochondrial transplantation using this technique has been demonstrated to rescue mitochondrial functions in mitochondrial diseases, including myoclonic epilepsy with ragged-red fibers (MERRF) [60,61]. The same method was further utilized in human breast adenocarcinoma cell lines MCF-7 and MDA-MB-231 [30]. Although the same cell types can take up mitochondria without the need of carriers [31], other diseased cells have lower spontaneous intake due to cytoskeletal disruption and can thus benefit from this method [12,62].

## 3. Metabolic and Phenotypic Consequences of Mitochondrial Transfer

### 3.1. Restoration of Basic Mitochondrial Functions in Cancer Cells

Mitochondrial transfer can have several consequences in recipient cells. Initial studies of mitochondrial transfer were performed in cells devoid of mitochondrial DNA by treatment with ethidium bromide (ρ0 cells). This resulted in the recovery of recipient cell mitochondrial activities due to the acquisition of whole mitochondria from donor cells. In 2006, Spees et al. [63] reported a decrease in extracellular lactate, decreased ROS, increased extracellular ATP, increased membrane potential and increased oxygen consumption after mitochondrial transfer between A549 ρ0 human adenocarcinoma cells and co-cultured MSCs or human skin fibroblasts, indicative of a complete restoration of mitochondrial activities. Similarly, 143B ρ0 osteosarcoma cells exhibited recovered mitochondrial functions via increased intracellular ATP and an increased oxygen consumption rate after co-culture with MSCs [23]. Using the same osteosarcoma model, an additional study described the uptake of mitochondria from Wharton’s jelly MSCs (WJMSCs) and the subsequent restoration of mitochondrial complexes and their associated functions [28]. To our knowledge, no study reported functional consequences for donor cells.

### 3.2. Cancer Cell Survival and Proliferation

Mitochondrial transfer has also been documented to affect recipient cell proliferation and survival. Accordingly, Elliot et al. [31] documented that the transfer of isolated mitochondria from normal breast epithelium MCF-12A to MCF-7 and MDA-MB-231 breast cancer cells and adriamycin-resistant NCI/ADR-Res ovarian cancer cells resulted in a decrease in proliferation of MCF-7 and NCI/ADR-Res cells and increased the sensitivity of MCF-7 cells to doxorubicin, paclitaxel and carboplatin. Assisted methods of mitochondrial transfer from healthy cells via a Pep-1-mediated mechanism further demonstrated impaired in vitro viability of MCF-7 breast cancer cells and in vivo suppression of tumorigenicity [30]. Interestingly, cell viability was unchanged after mitochondrial transfer in the non-malignant MCF-12A human breast cell line.

Although these studies present promising therapeutic outcomes, others have documented contradictory phenomena after mitochondrial transfer. For example, Caicedo et al. [26] noted that the transfer of mitochondria from MSCs to cancer cells led to enhanced mitochondrial functions (increased OXPHOS and ATP production), which led to increased proliferation and invasion phenotypes of these cells. Likewise, Marlein et al. [34] observed that mitochondrial transfer from BMSCs to primary myeloma cells led to an increase in their ATP production and proliferation rate both in vitro and in vivo. Wang and Gerdes [21] also observed a decrease in apoptosis in UV-stressed PC12 phaeochromocytoma cells after mitochondrial transfer when cultured with healthy cells, indicative of an increase in cancer cell survival.

### 3.3. Tumorigenesis and Tumor Progression

Building upon controlled mitochondrial transfer experiments, a systems approach to mitochondrial transfer has also been utilized to define key characteristics surrounding the tumor microenvironment and tumor development. In the mitochondria-deficient B16 ρ0 metastatic mouse melanoma cell line and 4T1 ρ0 mouse mammary cancer cell line, cancer cells were found to acquire mtDNA from cells in the tumor microenvironment, which resulted in the recovery of their mitochondrial respiration [27]. This effect was compounded in circulating cancer cells when compared to primary tumors, suggesting an adaptive response to progressively acquire mtDNA from the tumor microenvironment as part of cancer cell invasiveness. In a follow-up study, the same authors demonstrated that tumor formation was directly dependent upon the recovery of mitochondrial respiration [63]. The latter demonstration that the transfer of intact mitochondria from cancer-associated fibroblasts to PC3 human prostate cancer cells [39] and from highly metastatic to weakly metastatic mouse Lewis lung carcinoma cancer cells [64] also transferred metastatic capabilities indicates that mitochondria play a key role in cancer metastasis. Accordingly, either highly active TCA cycling or electron transport chain (ETC) bottlenecking were shown to promote cancer migration, invasion and metastasis through mtROS activating the transforming growth factor β (TGFβ) pathway [65].

Furthermore, in an AML model, cancer cells acquired mitochondria from stromal cells in the tumor microenvironment, thus improving their metabolic function through an increased production of ATP [29]. Finally, in a patient-derived organoid model using glioblastoma stem cells, mitochondrial transfer occurred between cells in both 2D and 3D cultures, indicating that this phenomenon also occurs in human brain tumors [40].

### 3.4. Chemoresistance

Several recent studies in cancer biology have documented the acquisition of chemoresistance after mitochondrial transfer. The first experiment to document this phenomenon was in 2013, when Pasquier et al. [22] observed that MCF-7 cancer cells acquiring mitochondria from epithelial cells gained resistance to doxorubicin. Since then, more recent studies have further explored the induction of chemoresistance after mitochondrial transfer. In Jurkat and ALL cells as donor cells and MSCs as recipient cells, mitochondrial transfer was shown to induce MSC-dependent chemoresistance to cytarabine and methotrexate through a reduction of ROS levels [36]. Likewise, mitochondrial transfer from MSCs to ALL cells induced chemoprotection from ROS-inducing cytarabine and daunorubicin therapies [35], confirming that the transfer of mitochondria from one cell type to another can generate chemoresistance in recipient cells.

According to these studies, ROS appear to be a central chemoresistance checkpoint after mitochondrial transfer. However, ROS can also stimulate mitochondrial transfer itself. Indeed, Marlein et al. [32] demonstrated that AML cells can increase their own ROS production by activating NADP(H) oxidase NOX2, which stimulated a mitochondrial transfer from BMSCs to AML cells both in vitro and in vivo. Conversely, NOX2 knockdown in AML cells reduced superoxide production, which resulted in decreased cellular uptake of mitochondria by AML cells and a reduced basal and maximal mitochondrial respiration. These results were replicated in vivo, indicating a pro-tumoral role of NOX2-driven mitochondrial transfer in AML [32]. Recently, a ROS-overload mediated mitochondrial transfer mechanism has been questioned. In a model using TASCs and primary glioblastoma cells [38], co-cultured cells showed a significant decrease in primary glioblastoma ROS levels compared to monocultures, as well as a significant increase in glycolysis. The authors concomitantly observed an unidirectional mitochondrial transfer from TASCs to primary glioblastoma cells. Therefore, identifying the precise nature of ROS, their subcellular origin and their local concentration is now needed to understand their roles in mitochondrial transfer.

## 4. Conclusions and Therapeutic Perspectives

This comprehensive review addressed intercellular mitochondrial transfer in cancer. It illustrated that cancer cells can acquire mitochondria from neighboring cells not only to repopulate an intact mitochondrial pool, but also to acquire phenotypic characteristics that represent gain of functions for tumors such as an increased rate of proliferation, enhanced migrative, invasive and metastatic capabilities and resistance to chemotherapies. Stemness could potentially be included to the list, as (cancer) stem cell mitochondrial metabolism most often differs from that of differentiated (cancer) cells [66]. While mtDNA encodes only 13 mitochondrial proteins in addition to 22 transfer RNAs and 2 ribosomal RNAs, SNPs, mutations, deletions and/or epigenetic changes (methylation, non-coding RNAs) could convey these traits long-term [67]. Conversely, gain of function mutations could fade away depending on the half-life of specific mitochondrial components that would directly or indirectly depend on the nuclear DNA expression of donor cells. To help elucidate these phenomena, as well as to increase knowledge about the persistence or resolution of heteroplasmy, studies examining mitochondrial kinetics would be required. 

This review also showed that cancer cells can transfer mitochondria to nonmalignant cells, which can be viewed as an additional mechanism to mitophagy for the clearance of damaged mitochondria. Little is known about their fate in the recipient cells. It is also not known if mitochondria acquired from different cell populations can exhibit differential effects in recipient cancer cells. To date, only one study [22] showed a common phenotype, chemoresistance, acquired after mitochondrial transfer from two different donor populations (MSCs and endothelial cells), suggesting that phenotypic acquisition may be independent of donor cell origins if the received mitochondria are healthy. To adequately address this question, further studies are needed where donor mitochondria from several cell types would be transferred to the same host cells under the same treatment and culture conditions.

This review also provides indications for future anticancer applications. Considering that several studies correlate mitochondrial transfer with chemoresistance [22,30,32,35,36] and with cancer cell recovery after treatment [23,28,63], a potential approach to prevent acquired chemoresistance would be to evaluate inhibition of mitochondrial transfer as an adjuvant treatment. Before full understanding of the process and identification of precise, druggable targets, this could be achieved indirectly by the use of taxanes or *Vinca* alkaloids that have the potential to partially inhibit mitochondrial transfer by inhibiting microtubule polymerization. It could also be noteworthy to investigate whether these treatments could restrict metastatic dissemination in an in vivo mouse setting where mitochondrial transfer was associated with a gain in migration and/or invasion [39,64].

M-sec, a TNT marker and regulator of TNT formation [45], has been proposed as a targetable inhibitor of mitochondrial transfer. While M-sec catalytic sites remain unknown, currently marketed TNF-α inhibitors (including those prescribed for auto-immune diseases such as rheumatoid arthritis) may indirectly reduce TNT formation, since M-sec is directly TNF-α inducible [68].

GJ proteins, such as Cx43, have been found to participate in mitochondrial transfer [58], but their ubiquitous expression in almost every tissue type in the body renders them unsuitable as a pharmacological target. Interestingly, Cx43 single point mutations causing alterations of specific phosphorylation sites have been reported [69], but it remains to be elucidated if TNT-specific phosphorylation sites on Cx43 exist. If so, kinases and/or phosphatases responsible for specific phosphorylation patterns may exhibit therapeutic promises once discovered.

Mitochondrial fission has never been studied in correlation with TNTs. Mitochondrial fission is a necessary step for mitophagy because smaller, non-elongated mitochondria are easier to be incorporated into autophagosomes. Fission may similarly be necessary for mitochondrial transfer, hypothetically easing mitochondrial migration along TNTs. If this hypothesis were true, mitochondrial fission inhibition would simultaneously target mitophagy and mitochondrial transfer, two promising anticancer approaches for which there is no current selective therapy.

## Figures and Tables

**Table 1:** Mitochondrial transfer involving cancer cells (2011–2021).

Cells	Mechanism of Mitochondrial Transfer	Mitochondrial Traffic	Functional Consequences in Recipient Cells	Type of Study	Reference
Rat PC12 phaeochromocytoma cells untreated ± UV-damage	TNTs— Membrane protrusions (MPs)	Bidirectional between healthy cells Unidirectional from healthy to UV-damaged cells	Rescue of UV-damaged from apoptosis	In vitro	[21]
Human stromal cells (endothelial and mesenchymal stem cells [MSCs]) and human ovarian and breast cancer cells	TNTs	Bidirectional (preference for endothelial to cancer cells)	Chemoresistance	In vitro	[22]
Human mitochondria-deficient (ρ0) 143B ρ0 osteosarcoma cells and MSCs	Suggested TNTs	Unidirectional from MSCs to 143B ρ0, but not to 143B cells	Restoration of mitochondrial functions	In vitro	[23]
Human laryngeal squamous cell carcinoma (LSCC)	TNTs	Not clarified	No documented consequences	In vitro In tissue	[24]
Human mesothelioma and benign mesothelial cell lines	TNTs	Bidirectional between malignant or between normal, not existing between malignant and normal.	No documented consequences	In vitro In tissue	[25]
Human MSCs and cancer cell lines	TNTs (artificial transfer and uptake by cancer cells in a not detailed way)	Bidirectional between MSCs and cancer cells, among MSCs but not among cancer cells. Artificial unidirectional from MSCs to cancer cell lines	Increased OXPHOS and ATP production. Increase invasion and proliferation.	In vitro	[26]
B16 ρ0 mouse metastatic melanoma and 4T1 ρ0 mouse metastatic breast cancer cells	Not clarified	Not clarified—acquisition of mtDNA from the microenvironment	Stepwise recovery of respiration and tumorigenicity	In vivo	[27]
Human 143B ρ0 osteosarcoma cells and human Wharton’s jelly MSCs (WJMSCs)	Not clarified	Unidirectional from WJMSCs to 143B ρ0	Restoration of respiratory complexes and function of cancer cells	In vitro	[28]
Human acute myelogenous leukemia (AML) cell lines, umbilical cord blood (CB) and MS-5 stromal cell line	Not clarified. Endocytosis has been suggested	Unidirectional from stromal cells to AML cells	Improved ATP production by AML cells Increased chemoresistance potential of AML cells	In vitro In vivo	[29]
Human MCF-7 and MDA-MB-231 breast adenocarcinoma cell lines and homoplasmic 143B osteosarcoma cells with wild-type mtDNA	Not clarified	Unidirectional from wild-type mtDNA cells to breast cancer cells	Inhibition of cell proliferation, induction of apoptosis and increased drug sensitivity	In vitro In vivo	[30]
Human MCF-12A mammary epithelial cell line, human MCF-7 and MDA-MB-231 breast cancer cell lines and drug-resistant NCI/ADR-Res cancer cell line	Artificial transfer. Mechanisms of uptake not clarified. Endocytosis suggested	Unidirectional from untransformed MCF-12A to malignant MCF-7, MDA-MB-231 and NCI/ADR-Res cells	Suppression of MCF-7 and NCI/ADR-Res cell proliferation. Increased drug sensitivity of MCF-7 cells.	In vitro	[31]
Human AML blasts and bone marrow stromal cells (BMSCs), healthy hematopoietic stem cells	TNTs	Unidirectional from BMSCs to AML blasts but not to healthy hematopoietic stem cells	Increased basal and maximum mitochondrial respiration and ATP production in AML blasts	In vitro In vivo	[32]
Human highly malignant T24 urothelial carcinoma cells and non-malignant RT4 urinary papillary urothelial cells	TNTs	Unidirectional from malignant to non-malignant cells	Enhanced non-malignant cell invasiveness	In vitro In vivo	[33]
Primary human multiple myeloma cells and cell lines, BMSCs	TNTs	Bidirectional	Increased ATP production and proliferation of multiple myeloma cells	In vitro In vivo	[34]
Human MSCs and acute lymphoblastic leukemia (ALL) cells	TNTs	Unidirectional from MSCs to ALL cells	Chemoprotection from ROS-induced therapy	In vitroIn vivo	[35]
Human MSCs, Jurkat cells and T-ALL cells	TNTs	Bidirectional, but mostly from Jurkat cells to MSCs and from T-ALL cells to MSCs	Chemoresistance of Jurkat and T-ALL cells	In vitro	[36]
Human 1321N1 astrocytoma cells, THP-1 monocytic leukemia cells and SH-SY5Y neuroblastoma cells	TNTs	Not clarified	α-synuclein aggregates as mitochondria transfer through TNTs	In vitro	[37]
Human tumor activated stromal cells (TASCs) and glioblastoma cells	TNTs, EVs and cannibalism	Unidirectional from TASCs to primary glioblastoma cells	Chemoresistance, radioresistance, increased proliferation in glioblastoma cells	In vitro	[38]
Human PC3 prostate cancer cells and cancer-associated fibroblasts (CAFs)	TNTs	Unidirectional from CAFs to PC3 cancer cells	Increased migratory and metastatic abilities of prostate cancer cells	In vitro	[39]
2D and 3D primary glioblastoma (GBM) stem cells	TNTs	Not clarified	Differential mitochondria transferred after irradiation regimen	In vitro	[40]

## Data Availability

Not applicable.
